# A Novel Position Compensation Scheme for Cable-Pulley Mechanisms Used in Laparoscopic Surgical Robots

**DOI:** 10.3390/s17102257

**Published:** 2017-09-30

**Authors:** Yunlei Liang, Zhijiang Du, Weidong Wang, Lining Sun

**Affiliations:** State Key Laboratory of Robotics and System, Harbin Institute of Technology, 2 Yikuang Street, Harbin 150080, China; liangyunlei@stu.hit.edu.cn (Y.L.); duzj01@hit.edu.cn (Z.D.); lnsun@hit.edu.cn (L.S.)

**Keywords:** cable-pulley system, backlash hysteresis, position compensation, support vector machine

## Abstract

The tendon driven mechanism using a cable and pulley to transmit power is adopted by many surgical robots. However, backlash hysteresis objectively exists in cable-pulley mechanisms, and this nonlinear problem is a great challenge in precise position control during the surgical procedure. Previous studies mainly focused on the transmission characteristics of the cable-driven system and constructed transmission models under particular assumptions to solve nonlinear problems. However, these approaches are limited because the modeling process is complex and the transmission models lack general applicability. This paper presents a novel position compensation control scheme to reduce the impact of backlash hysteresis on the positioning accuracy of surgical robots’ end-effectors. In this paper, a position compensation scheme using a support vector machine based on feedforward control is presented to reduce the position tracking error. To validate the proposed approach, experimental validations are conducted on our cable-pulley system and comparative experiments are carried out. The results show remarkable improvements in the performance of reducing the positioning error for the use of the proposed scheme.

## 1. Introduction

Robotic minimally invasive surgery (RMIS), which is famous for its high success rate in complex minimally invasive surgery, is one of the most advanced technologies in surgical fields. Compared with the traditional minimally invasive treatment, RMIS allows better hand-eye coordination, field of vision, accuracy and stability [1]. RMIS can help surgeons perform surgeries with maximum precision and minimal deep invasion inside a human body, and give surgeons dexterous instrument control [2,3,4]. Due to these advantages, RMIS can elevate the rates of surgical success and reduce the pain of patients, the need for medication and the duration of hospital stays [5,6]. Although RMIS has lots of advantages, increasing the operation precision is still necessary. Surgical robots do not get tired as people do, but they are not intelligent enough to judge whether an action is beneficial for patients, and they can only obey surgeons’ instructions and follow computer programs to perform operations. Therefore, robots cannot create self-corrected or positive changes when the robotic end-effector has motion errors which will reduce position accuracy. Lower positioning accuracy directly leads to the result that the robotic end-effector cannot reach the specified location, which increases the surgical risk. As the end-effector of the laparoscopic surgical robot is operated inside the patient’s body, transmitting surgical operations precisely is very important during surgery. If there is any position error, it is extremely dangerous because the end-effector may damage the patient’s tissues or organs and even threaten the patient’s life. This situation is unacceptable and also determines the significance of guaranteeing the kinematic accuracy of the robotic end-effector.

**Figure 1:**
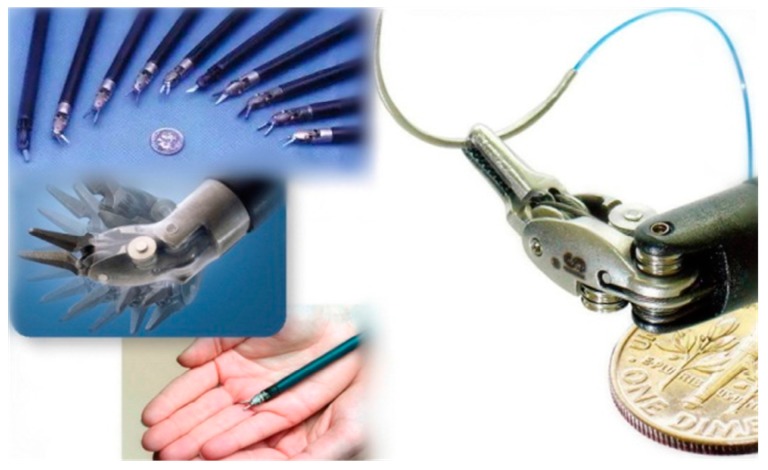
Some surgical robot instruments.

**Figure 2:**
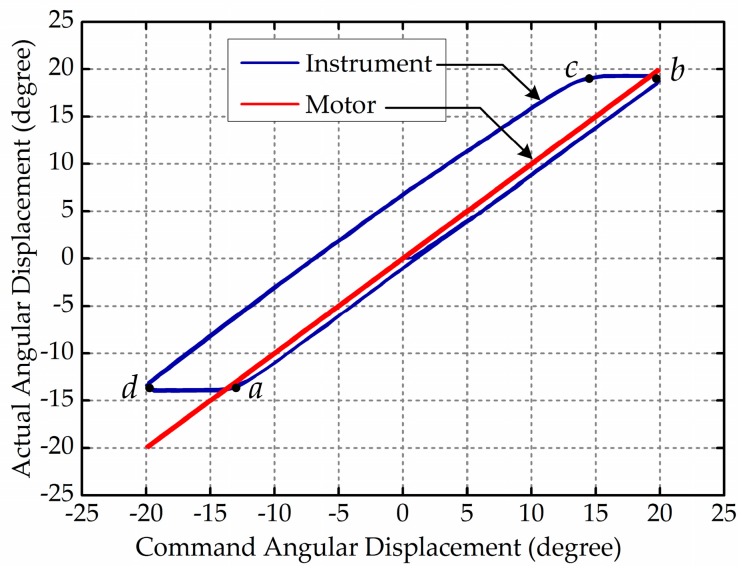
The relationship between actual and command angular displacement: segments *ab* and *cd* represent backlash hysteresis; segments *bc* and *ad* represent normal movement.

The surgical robot for an endoscope operation consists of micro instruments which perform the operation directly inside the patient’s body as an extension of the surgeon’s hands, as shown in Figure 1. Figure 1 shows the size proportion of the end-effector and pulleys, the wrapping pattern of the cable and the motion pattern of the end-effector. Micro surgical instruments need to be extremely flexible, reliable and precise to ensure smooth operation, therefore most of them adopt the cable-pulley mechanism [7,8,9,10] or tendon-sheath mechanism [11,12,13]. The cable-pulley system is similar to the tendon-sheath system, both of which are affiliated with the flexible-wire driven system and widely applied in surgical robots. Compared to a general rigid power transmitter like a shaft, gear or chain, the flexible-wire driven system has more advantages such as being lightweight, small and highly flexible. In addition, because it does not need any oil or lubricants, the flexible-wire driven system is more convenient to disinfect and more effective at reducing the risk of infection. However, there are many undesirable nonlinear problems in flexible-wire transmission, such as dead zones, backlash hysteresis and direction-dependent behavior. Among these nonlinear problems, the effects of backlash hysteresis on the positioning accuracy of the surgical robot are dramatic, as shown in Figure 2.

Backlash hysteresis for surgical robots mainly originates from two causes: the clearance of the transmission system which objectively exists, and the conversion between the flexible-wire’s slack side and tight side when the motor moves in a different direction. Data shows that the positioning error is about 2–6 degrees for each degree of freedom (DOF) in a surgical robot’s end-effector, which is fatal and unacceptable during surgery. Existing studies mainly concentrate on the characteristics of flexible-wire transmission and transmission models under specific assumptions are designed to solve certain nonlinear problems. Kaneko et al. [14,15] discussed the input-dependent stability observed during torque control experiments using the first joint of the Darmstadt-HAND. They designed a transmission model under the assumptions that the pretension is large enough to avoid slacking, the sheath curvature is fixed, and the friction between tendon and sheath follows the Coulomb-based friction model. Based on experiments, they formulated model equations characterizing the input dependency and explored the stability by using the technique of sinusoidal input describing functions (SIDF). Agrawal et al. [16,17] assumed the environmental load can be described by specific expressions and took the physical limitations of the actuator into consideration. They developed a mathematically distributed model for the transmission characteristics in cable-conduit mechanisms, which described nonlinear behaviors of the dual tendon-sheath like backlash. Wu et al. [18] assumed that the pretension in the tendon is large enough to prevent any system slacking due to tendon elasticity, and the curvature distribution along the longitudinal direction is considered to remain the same for each tendon during the operation of the robot joint. They proposed a general mathematical double-tendon-sheath transmission model suitable for arbitrary types of load conditions. Do et al. [19,20,21,22,23] presented a dynamic friction model of the tendon-sheath system based on several assumptions, such as fixed initial pretension and fixed accumulated curve angles. The proposed model can reduce the number of model parameters compared to the original asymmetric Bouc-Wen model and enhance the tracking performance of the tendon-sheath. Sun et al. [24,25,26] modeled the tendon elongation in a flexible tendon-sheath system under some assumptions and proposed a system modeling approach for motion compensation. They analyzed position errors according to system backlash and environment load, and the proposed motion compensation strategy can enhance the performance of position control. Qi et al. [27] assumed that the angle of the axial rotation of a winch, a movable pulley or a fixed pulley can be expressed as a quintic polynomial. They developed an accurate mathematical model and realized a dynamic simulation of a cable-pulley system. They formulated the governing equations based on the principle of virtual power with the reduction technique of DOFs, and the motions of each pulley and each segment of cable can be worked out by solving the dynamic equations. Xue et al. [28] presented a tension and displacement transmission model of a cable-pulley system in the end-effector of a laparoscopic surgical robot. They assumed that the steel cable has elasticity and bending stiffness during the transmission process. Furthermore, they neglected the stretching acceleration in the whole steel cable and centrifugal acceleration in the contact zone with pulleys when the grasper changes direction suddenly. Results from their experiments showed substantial improvements in the performance of position tracking errors for the use of the proposed algorithm. The schemes proposed in the existing studies can greatly reduce the positioning error caused by backlash hysteresis. However, these approaches still have limitations, such as specific assumptions used during the modeling process. In general, the fewer assumptions we make, the more precise the transmission model is, and subsequently the more complex the transmission model is. Besides, to build a transmission model which completely reflects the real system is impossible. Therefore, researchers should provide a trade-off between the precision and complexity of the transmission model, and have better modeling ability. Meanwhile, the transmission model will change if the surgical instrument is replaced or the mechanical structure of the surgical instrument is changed, which results in rebuilding the transmission model frequently.

To eliminate the effects of backlash hysteresis, we need to know actually when the robotic end-effector begins to move. Fortunately, lots of useful information is contained in the motor current which largely reflects the running state of the motor [29,30], and this information can be used to analyze the movement of the robotic end-effector. Taking the above into account, a new position compensation method for the cable-pulley system used in surgical robots is presented in this paper. Useful information is extracted from the motor current based on a support vector machine (SVM), which can recognize and classify different movement stages (backlash hysteresis and normal movement) of the robotic end-effector. After the movement stages classification, the positioning error resulting from backlash hysteresis can be compensated. Theoretical analysis and experimental results show that the position compensation method presented in this paper can efficiently eliminate the backlash hysteresis nonlinearities and improve the positioning precision of the robotic end-effector. Compared with traditional methods, this approach is simpler and has better versatility.

In the next section, this paper analyzes the motor current of surgical robots and introduces the position compensation method based on SVM. In the third section, the position compensation method is proven by experimental testing. Lastly, this paper makes a conclusion.

## 2. The Position Compensation Scheme 

**Figure 3:**
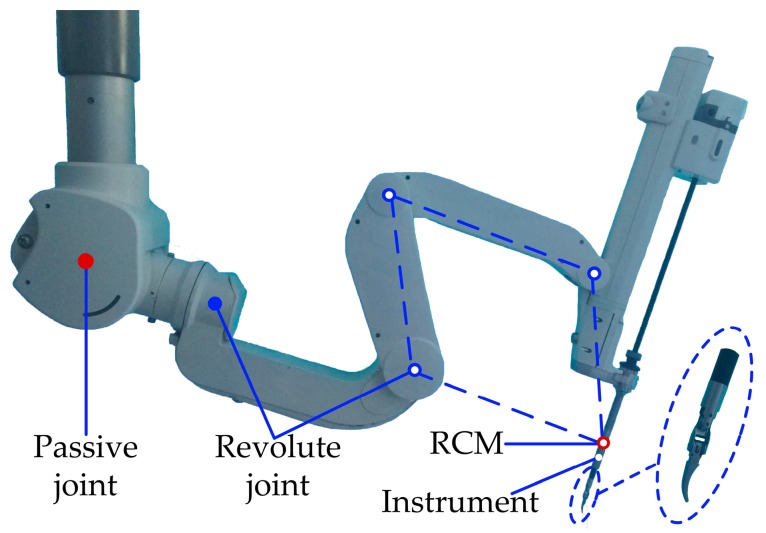
The robotic arm and surgical instrument studied in this paper.

In [1,28,31], the robotic arm and surgical instruments studied in this paper are introduced. The robotic arm carried a certain surgical instrument shown in Figure 3. The robotic arm has three DOFs and is easy to set up. The surgical instrument has four DOFs and can complete the surgical operation inside the patient body effectively. The cable-pulley mechanisms used in the surgical instrument are illustrated in Figure 3. In this section, the kinetic model of the cable-pulley system is established to analyze the drive torque of the motor. Then the movement stages of the end-effector are classified by using SVM and a feedforward position compensation control algorithm is introduced.

### 2.1. Kinetic Analysis for the Cable-Pulley Mechanism

**Figure 4:**
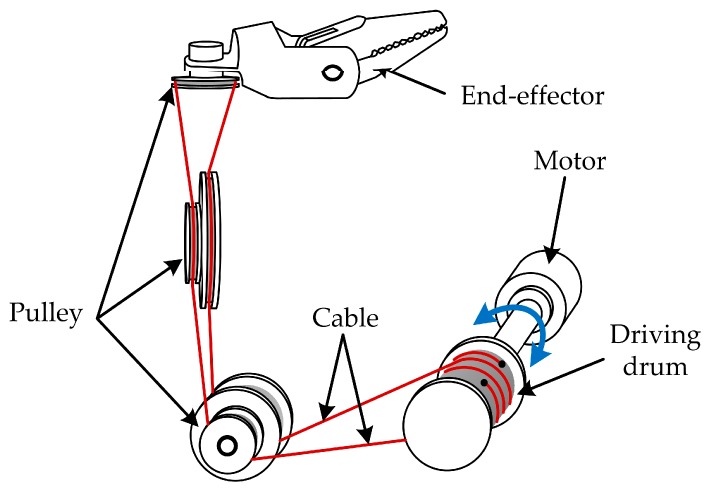
The cable-pulley system studied in this paper.

**Figure 5:**
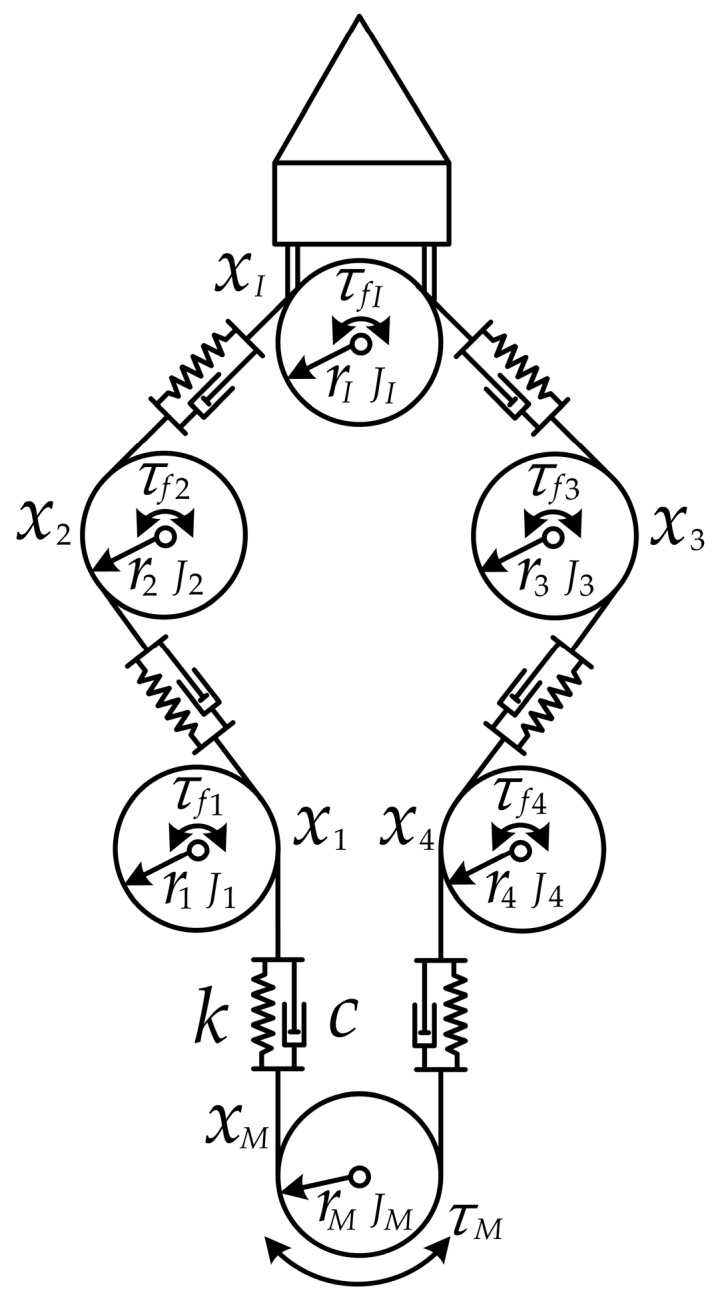
The simplified physical model of the cable-pulley system.

Figure 4 shows the cable-pulley system studied in this paper. The pulleys installed on the same shaft can rotate independently and the end-effector rotates reciprocally as the motor rotates in different directions, as is shown below. The kinetic analysis of the cable-pulley mechanism is only used to find the analysis object which can reflect the movement of the end-effector and will not be used in the design of the position compensation scheme. In the cable-pulley transmission system, the bending and torsional moments of the cable hardly influence the kinetic characteristics of the transmission system. Meanwhile, the references [14,15,18] show that the bending and torsional moments of the cable can be ignored, even though we want to build an accurate transmission model for the cable-pulley mechanism. Therefore, in order to simplify the kinetic analysis, the bending and torsional moments of the flexible cable which is abstracted into the spring damping model are neglected. The simplified physical model is illustrated in Figure 5. According to the simplified physical model of the cable-pulley mechanism, we can analyze the kinetic characteristic of each pulley easily.

This paper analyzes the movement of each pulley separately, so the general equations of motion for the cable-pulley mechanism can be formulated as follows:

(1) $$\left[K\right]\left\{\begin{array}{l} x_{1}\\ x_{2}\\ x_{3}\\ x_{4}\\ x_{I}\\ x_{M} \end{array}\right\}+\left[C\right]\left\{\begin{array}{l} {\dot{x}}_{1}\\ {\dot{x}}_{2}\\ {\dot{x}}_{3}\\ {\dot{x}}_{4}\\ {\dot{x}}_{I}\\ {\dot{x}}_{M} \end{array}\right\}+f=\left[J\right]\left\{\begin{array}{l} {\ddot{x}}_{1}\\ {\ddot{x}}_{2}\\ {\ddot{x}}_{3}\\ {\ddot{x}}_{4}\\ {\ddot{x}}_{I}\\ {\ddot{x}}_{M} \end{array}\right\},$$

where [K], [C] and [J] are the coefficient matrix; $x_{1}$, $x_{2}$, $x_{3}$, $x_{4}$, $x_{M}$ and $x_{I}$ are the displacement of the corresponding position. Through the derivation, [K], $f^{T}$, [C] and [J] can be expressed as follows:

(2) $$\left[K\right]=\left[\begin{array}{cccccc} -2k & k & 0 & 0 & 0 & k\\ k & -2k & 0 & 0 & k & 0\\ 0 & 0 & -2k & k & k & 0\\ 0 & 0 & k & -2k & 0 & k\\ 0 & k & k & 0 & -2k & 0\\ k & 0 & 0 & k & 0 & -2k \end{array}\right],$$

(3) $$f^{T}=\left\{\begin{array}{llllll} \frac{\tau_{f1}}{r_{1}} & \frac{\tau_{f2}}{r_{2}} & \frac{\tau_{f3}}{r_{3}} & \frac{\tau_{f4}}{r_{4}} & \frac{\tau_{fI}}{r_{I}} & \frac{\tau_{fM}}{r_{M}} \end{array}\right\},$$

(4) $$\left[C\right]=\left[\begin{array}{cccccc} -2c & c & 0 & 0 & 0 & c\\ c & -2c & 0 & 0 & c & 0\\ 0 & 0 & -2c & c & c & 0\\ 0 & 0 & c & -2c & 0 & c\\ 0 & c & c & 0 & -2c & 0\\ c & 0 & 0 & c & 0 & -2c \end{array}\right],$$

(5) $$\left[J\right]=\left[\begin{array}{cccccc} \frac{J_{1}}{r_{1}^{2}} & 0 & 0 & 0 & 0 & 0\\ 0 & \frac{J_{2}}{r_{2}^{2}} & 0 & 0 & 0 & 0\\ 0 & 0 & \frac{J_{3}}{r_{3}^{2}} & 0 & 0 & 0\\ 0 & 0 & 0 & \frac{J_{4}}{r_{4}^{2}} & 0 & 0\\ 0 & 0 & 0 & 0 & \frac{J_{I}}{r_{I}^{2}} & 0\\ 0 & 0 & 0 & 0 & 0 & \frac{J_{M}}{r_{M}^{2}} \end{array}\right],$$

where *k* is the spring stiffness; *c* is the damping coefficient; $\tau_{f1}$, $\tau_{f2}$, $\tau_{f3}$, $\tau_{f4}$ and $\tau_{fI}$ are the friction moment; $J_{1}$, $J_{2}$, $J_{3}$, $J_{4}$ and $J_{I}$ are the moment of inertia of the pulleys; $r_{1}$, $r_{2}$, $r_{3}$, $r_{4}$ and $r_{I}$ are the radius of the pulleys; $\tau_{M}$, $J_{M}$ and $r_{M}$ represent the drive torque of the motor, the moment of inertia of the drive shaft and the radius of the drive shaft. According to Equation (1), after eliminating some parameters, the drive torque of the motor can be expressed as follows:

(6) $$\tau_{M}=\frac{J_{M}{\ddot{x}}_{M}}{r_{M}}+(\sum_{i=1}^{4}\frac{J_{i}{\ddot{x}}_{i}+\tau_{fi}r_{i}}{r_{i}{}^{2}}+\frac{J_{I}{\ddot{x}}_{I}+\tau_{fI}r}{r_{I}{}^{2}})\cdot r_{M}$$

The end-effector stays in the stage of normal movement only if the motor torque is high enough to overcome the friction moments, so the critical value of motor torque can be expressed as follows:

(7) $$\tau_{M}=(\frac{\tau_{f1}}{r_{1}}+\frac{\tau_{f2}}{r_{2}}+\frac{\tau_{fI}}{r_{I}}+\frac{\tau_{f3}}{r_{3}}+\frac{\tau_{f4}}{r_{4}})\cdot r_{M}$$

The acceleration terms in Equation (6) do not affect the critical value of motor torque and the transmission system can only overcome the friction moments when $\tau_{M}$ satisfies Equation (7). The value of $\tau_{M}$ is influenced by the acceleration of each pulley when the end-effector moves normally. According to the analysis above, the value of $\tau_{M}$ can be expressed as follows when the end-effector moves normally and when it overcomes the friction moment:

(8) $$\tau_{MN}=\frac{J_{M}{\ddot{x}}_{M}}{r_{M}}+(\sum_{i=1}^{4}\frac{J_{i}{\ddot{x}}_{i}+\tau_{fi}r_{i}}{r_{i}{}^{2}}+\frac{J_{I}{\ddot{x}}_{I}+\tau_{fI}r}{r_{I}{}^{2}})\cdot r_{M},$$

(9) $$\tau_{MT}=(\frac{\tau_{f1}}{r_{1}}+\frac{\tau_{f2}}{r_{2}}+\frac{\tau_{fI}}{r_{I}}+\frac{\tau_{f3}}{r_{3}}+\frac{\tau_{f4}}{r_{4}})\cdot r_{M},$$

where $\tau_{MT}$ is the threshold value of $\tau_{M}$ and the end-effector will not move until $\tau_{M}$ > $\tau_{MT}$, $\tau_{MN}$ is the value of $\tau_{M}$ when the end-effector moves normally. It is important to note that the deduction process of $\tau_{MT}$ and $\tau_{MN}$ is not mathematically strict, e.g., if $\tau_{f4}\gg$ ($\tau_{f1}$ + $\tau_{f2}$ + $\tau_{f3}$ + $\tau_{fI}$) or when $\tau_{f4}$ tends to be infinite, $x_{4}$ will be equal to 0, so $J_{4}{\ddot{x}}_{4}$ and $\tau_{f4}$ in $\tau_{MT}$ and $\tau_{MN}$ will disappear. However, $\tau_{f1}$, $\tau_{f2}$, $\tau_{f3}$, $\tau_{f4}$ and $\tau_{fI}$ are quite close in value and the errors of Equation (8) and Equation (9) are very small, so the derivation of $\tau_{MT}$ and $\tau_{MN}$ are reasonable. The research in this paper does not need the precise value of $\tau_{M}$ because there is no need to design a transmission model for the cable-pulley system, which is one of the advantages of this work.

### 2.2. Classification of the End-effector’s Movement Stages

**Figure 6:**
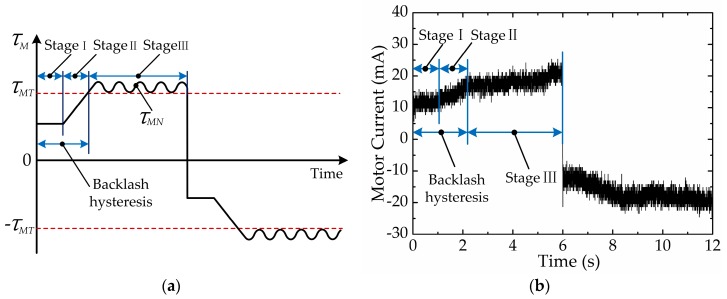
Varying patterns of motor driving moment and motor current: (**a**) varying patterns of motor driving moment; (**b**) varying patterns of motor current.

According to the discussion above, the moving processes of the cable-pulley system can be divided into three stages, as shown in Figure 6a. In the first stage, the motor begins to move, while the driving force of the motor has not yet transferred to the cable-pulley system because of the clearance of the transmission system. Since the motor has not yet driven the load, the value of $\tau_{M}$ is small. In the second stage, the motor begins to transfer torque to the cable-pulley system, and it stretches one side of the cable and slacks the other side of the cable at the same time. In this stage, the value of $\tau_{M}$ increases constantly with $\tau_{M}\leq \tau_{MT}$ and $\tau_{M}{=\tau }_{MT}$ at the end of this stage. The end-effector remains still at its original position even if the motor keeps moving during the first two stages, which is the backlash hysteresis phenomenon. In the third stage, the end-effector begins to move at the moment $\tau_{M}{=\tau }_{MN}$. In general, stage I and stage II belong to backlash hysteresis and stage III belongs to normal movement. We can distinguish different movement stages based on the changes of $\tau_{M}$. When the motor rotates in the opposite direction, $\tau_{M}$ has a similar variation trend and can also be divided into three movement stages. The only difference is the value of $\tau_{M}$, which is positive when the motor rotates in a forward direction and negative when the motor reverses. Meanwhile, there exists a linear relationship between the motor torque and current during normal operating, which can be expressed as follows:

(10) $$\tau_{M}=C_{t}I_{M},$$

where $C_{t}$ is the motor torque constant and $I_{M}$ is the motor current. As revealed by Equation (10), $\tau_{M}$ and $I_{M}$ have the same variation regularity, so we can also distinguish different movement stages based on the value of $I_{M}$ as illustrated in Figure 6b.

**Figure 7:**
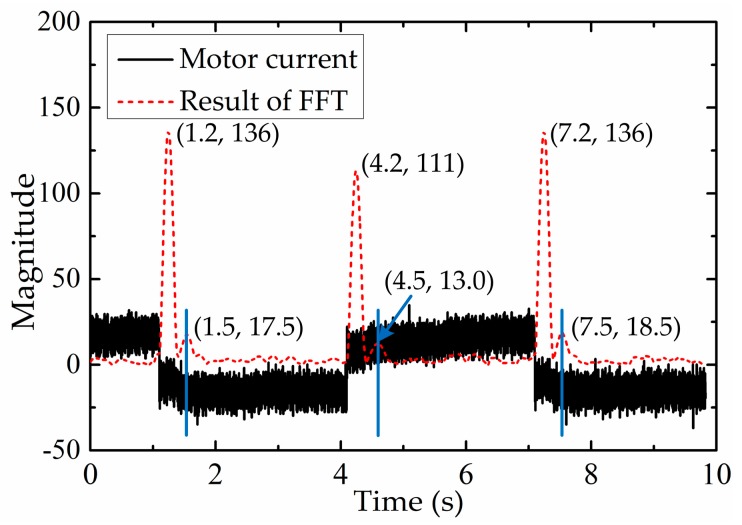
The result of FFT for the first non-zero frequency.

In order to obtain the movement condition of the end-effector, it is necessary to extract information from the motor current. Since fast Fourier transform (FFT) can provide the spectrum information of the motor current quickly and effectively, it is used to extract the implicit information from the motor current. Then the movement stages of the end-effector can be distinguished. In this paper, the motor current signal is sampled in chronological order and stored in a subset which has a fixed size. When a new current value is sampled, the subset will be modified by ‘shifting forward’, i.e., excluding the first current value of the data series and including the new current value in the subset. After the subset is modified, FFT will be operated on the data series in the subset. The data processing method above can guarantee the real-time performance of the calculation. The result of FFT for the first non-zero frequency is shown in Figure 7. It is evident from Figure 7 that there are two types of extreme values in the results of FFT for the first non-zero frequency. The larger extreme values such as (1.2, 136), (4.2, 111) and (7.2, 136) are caused by current reversal which corresponds to the motor reversing. The smaller extreme values such as (1.5, 17.5), (4.5, 13.0) and (7.5, 18.5) are caused by current variation which corresponds to the change of movement stage. Among these values, the smaller extreme values appear at the boundary between stage II and stage III. Through the analysis above, we can conclude that the FFT’s result of the motor current can be used to distinguish the movement stages of the end-effector, and then distinguish backlash hysteresis and normal movement. However, the result of FFT varies from the motor current which is influenced by the movement of the end-effector and external interference. In order to improve the accuracy and robustness of the movement stage division, a machine learning algorithm (SVM) is used to classify the movement stage of the end-effector. Machine learning explores the study and construction of algorithms that can learn from data and make predictions according to data. These algorithms are used more and more widely in the surgical field, e.g., in surgical gesture recognition [32], surgical outcome prediction [33], surgical expertise classification [34], kinematic control [35], surgical tool detection and tracking [36], etc. Because of the accuracy and efficiency of the classification effect, SVM can satisfy the requirement of movement stage classification in this paper.

### 2.3. The Position Compensation Scheme for the Cable-Pulley System Used in the End-effector

**Figure 8:**
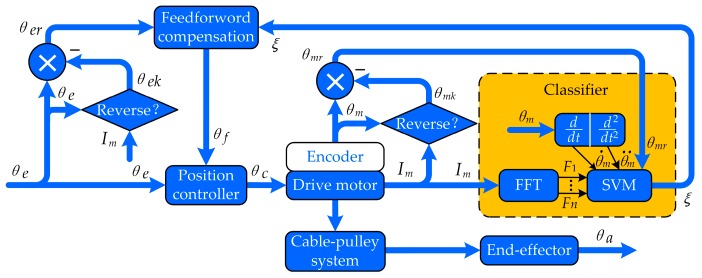
The position compensation control scheme based on SVM.

Since the backlash hysteresis of a cable-pulley system has great influence on the trajectory tracking performance of the robotic end-effector, it is essential to achieve the precise position control of the end-effector. The positioning error caused by the backlash characteristics should be compensated and a scheme of position compensation control based on SVM developed, as shown in Figure 8. In Figure 8, $\theta_{e}$, $\theta_{c}$ and $\theta_{f}$ represent the expected angular displacement, command angular displacement and compensating angular displacement, respectively; $\theta_{m}$ is the angular displacement obtained by the encoder of the motor; $\theta_{mk}$ and $\theta_{ek}$ respectively represent the value of $\theta_{m}$ and $\theta_{e}$ when the motor current is reversing, and $\theta_{mk}$ and $\theta_{ek}$ only change their value at the reversing moment; $\theta_{mr}$ is the angular displacement of the motor relative to the current reversing point as is expressed in Equation (11); $\theta_{er}$ is the expected angular displacement relative to the current reversing point as is expressed in Equation (12); $I_{m}$ is the motor current sampled in the control system; $\theta_{a}$ is the actual angular displacement of the end-effector; $F_{1}\cdots F_{n}$ represent the features extracted from the result of FFT; *ξ* represents the movement stage classified by SVM, and *ξ* could be 1, −1, or 0, representing normal movement in the positive direction, normal movement in the negative direction and backlash hysteresis, respectively. The command angular displacement is equal to the sum of the expected angular displacement and the compensating angular displacement, which can be expressed as Equation (13). The information extracted from the FFT result, such as the rotating velocity of the motor, the rotating acceleration of the motor and the angular displacement of the motor relative to the current reversing point, is provided to SVM as features. Then SVM is used to classify the movement stage of the end-effector, and the classification result is used to calculate the compensating angular displacement. Through the analysis of the movement stage above, the compensating angular displacement is equal to $\theta_{er}$ when the motor rotates in a forward direction and $-\theta_{er}$ vice versa. In the normal movement process of the end-effector, the compensating angular displacement is constant, i.e., $\theta_{f}\left(t\right){=\theta }_{f}\left(t^{-1}\right)$, so the compensating angular displacement can be expressed as Equation (14). According to Equation (14), the premise of position compensation is that the value of *ξ* can be obtained accurately, so the key technology of this paper is to design an efficient and accurate classifier.

(11) $$\theta_{mr}(t)=\theta_{m}(t)-\theta_{mk}(t)$$

(12) $$\theta_{er}(t)=\theta_{e}(t)-\theta_{ek}(t)$$

(13) $$\theta_{c}(t)=\theta_{e}(t)+\theta_{f}(t)$$

(14) $$\theta_{f}(t)=\{\begin{array}{llll} \theta_{er}(t)\cdot \mathrm{sgn}(\mathrm{current}) & & & \xi =0\\ \theta_{f}(t^{-1}) & & & \xi =1,-1 \end{array}$$

## 3. Experiment and Results

In this section, the proposed position compensation scheme is verified by experiments on our cable-pulley system. Firstly, an experimental platform is set up and the experimental data is collected. Then a classifier based on SVM is trained to guarantee the real-time capability and accuracy of the movement stage classification. Finally, in order to demonstrate the effectiveness of the proposed position compensation scheme, some comparative experiments on cable-pulley systems with and without position compensation are performed.

### 3.1. Experiment Setup

**Figure 9:**
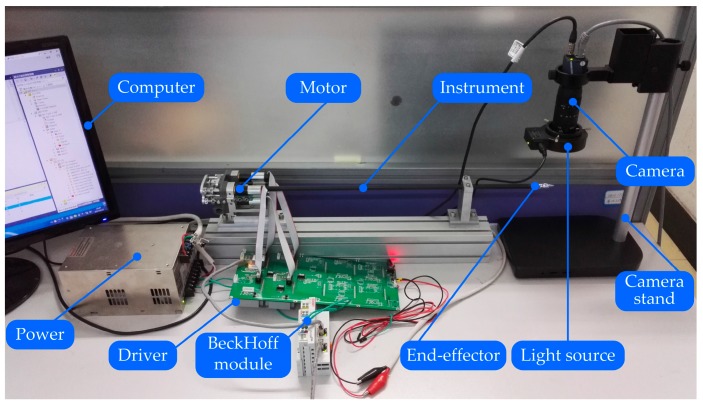
Photo of experimental setup.

**Figure 10:**
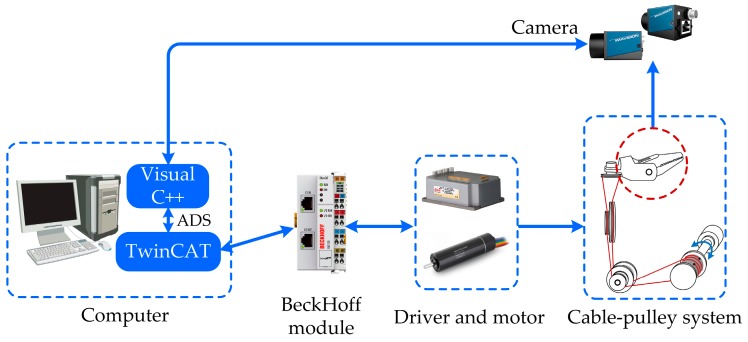
Introduction of the experiment system.

Based on the mentioned analysis, a dedicated experimental platform has been established to validate the position compensation scheme studied in this paper, as shown in Figure 9. Figure 10 shows the experimental system. The Maxon DC motor is utilized to connect the driving drum, which is actuated by the Elmo driver. The control of motion and collection of data are executed by TwinCAT. The BeckHoff module uses the EtherCat protocol to communicate with the computer and driver. In order to measure the actual angular displacement of the end-effector, the industrial digital camera MER-200-20GM/GC18 is used to capture images. The image is collected and processed in real-time by the software Visual C++ in the computer. The TwinCAT and Visual C++ use the ADS protocol to communicate with each other.

### 3.2. Training the Classifier and Classifying the Movement Stage in Real-Time

**Figure 11:**
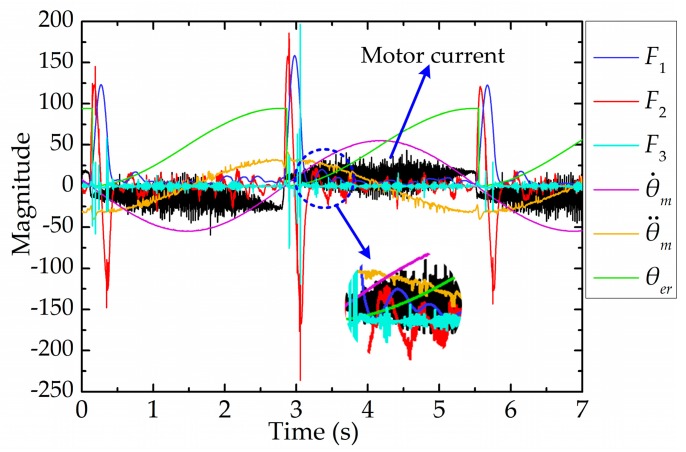
Initial selected features for SVM.

In order to design an efficient and accurate classifier, firstly, we needed to select the appropriate features for SVM. Several features were initially selected in this paper, such as the amplitude of the FFT’s result, the first-order derivative of the FFT’s result, the second-order derivative of the FFT’s result, the rotating speed of the motor (${\dot{\theta }}_{m}$), the rotating acceleration of the motor (${\ddot{\theta }}_{m}$) and the expected angular displacement relative to the current reversing point ($\theta_{er}$). Conveniently, $F_{1}$, $F_{2}$ and $F_{3}$ are used in this paper to represent the amplitude of the FFT’s result, the first-order derivative of the FFT’s result and the second-order derivative of the FFT’s result, respectively. In this paper, the extracted features are meaningful. $F_{1}$ reflects the magnitude of the FFT’s result, $F_{2}$ reflects whether or not the FFT’s result reaches an extreme value and $F_{3}$ reflects whether the FFT’s result is convex or concave. ${\dot{\theta }}_{m}$, ${\ddot{\theta }}_{m}$ and $\theta_{er}$ reflect the kinetic characteristic of the end-effector. These initial selected features are shown in Figure 11, where the expected angular displacement ($\theta_{e}$) is a sinusoidal wave trajectory.

In this paper,$\left\{\left(x_{j}^{\left(i\right)}{,y}^{\left(i\right)}\right);\;i=1,\;\cdots ,\;m,\;j=1,\;\cdots ,\;n\right\}$—is used to denote a training set; $x_{j}^{\left(i\right)}$ is the *j*-th feature of the *i*-th training example; $y^{\left(i\right)}$ is the label of the training example and is used to denote the movement stage of the end-effector. It is possible that only parts of the initially selected features are relevant to the learning task. Therefore, a heuristic method is adopted to choose a feature subset, which calculates the mutual information between $x_{j}$ and *y*. The calculation formula of the mutual information can be expressed as follows:

(15) $$\mathrm{MI}(x_{j},\;y)=\sum_{x_{j}}\sum_{y}p(x_{j},y)\log\frac{p(x_{j},y)}{p(x_{j})p(y)}$$

where ${\mathrm{MI}(x}_{j},\;y)$ is the mutual information between $x_{j}$ and y, reflecting the relevance between each feature $x_{j}$ and the class labels *y*; ${p(x}_{j},\;y)$, ${p(x}_{j})$ and p(y) are the probability of the corresponding event and can all be estimated according to the training examples. After calculation is complete, we simply pick the *k* features with the larger ${MI(x}_{j},\;y)$. In this study, 200,000 training examples are collected to train SVM, and the mutual information of each feature can be calculated according to these training examples. The results of the mutual information are listed in Table 1.

We can see that ${\dot{\theta }}_{m}$ and ${\ddot{\theta }}_{m}$ have minimal influence on the classification task from Table 1, so ${\dot{\theta }}_{m}$ and ${\ddot{\theta }}_{m}$ are removed from the training set. According to Equations (4) and (5), ${\dot{\theta }}_{m}$ is not relevant to $\tau_{MT}$ and $\tau_{MN}$, which explains the reason why the mutual information of ${\dot{\theta }}_{m}$ is minimal. It is worth pointing out that ${\ddot{\theta }}_{m}$ is not used as a classification feature in this study, although there is little difference between the mutual information value of ${\ddot{\theta }}_{m}$ and other features. Because ${\ddot{\theta }}_{m}$ is of high volatility, it is inappropriate to reflect the true value of the motor acceleration and is small in real surgical procedures for guaranteeing stability and safety of operation. After the classifying features are determined, 30% of the examples are randomly selected as the evaluation set, and the remaining 70% of the examples are the training set. By testing on the evaluation set that the SVM is not trained on, we can obtain a better estimate of true generalization error. Before training the classifier, the examples in the training set are standardized and RBF is chosen as the kernel function of SVM. SVM is trained on the training set and the parameters of SVM are searched heuristically. After the selection and optimization of the SVM parameters, the SVM is tested on the evaluation set. The prediction error rate of the classification for the evaluation set and the training set are listed in the Table 2. For convenience, notation Train: B→N is used to denote that the backlash hysteresis stage is classified as the normal movement stage on the training set, and notation Test: B→N is used to denote that the backlash hysteresis stage is classified as the normal movement stage on the evaluation set, and vice versa.

**Figure 12:**
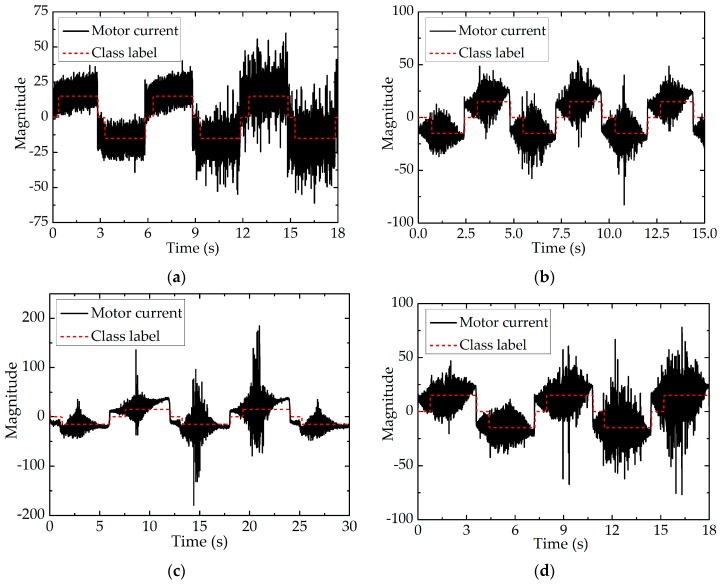
The classification effect of the movement stage when the motor runs in different motion patterns: (**a**) Uniform motion; (**b**) Uniform accelerated motion; (**c**) Variable accelerated motion; (**d**) Sinusoidal wave motion.

From Table 2, we can see that the classification error rate of SVM is very low, which can meet the accuracy requirements of movement stage classification. The results in Table 2 indicate the design of classifier is reasonable. In order to classify the movement stage in real time, the trained SVM algorithm is written into the control program. Numerous experiments demonstrate that the classification time of SVM classifier is less than 30 ms, which can meet the real-time requirements of movement stage classification. Figure 12 shows the classification effect of the movement stage when the motor runs in different motion patterns.

Figure 12 shows the classification effect of the movement stage when the motor runs in different motion patterns. In order to display the classification of the end-effector’s movement stage more clearly, ξ (the output of the classifier) is magnified 15 times as the value of the class label. Therefore, the value of the class label could be 15, −15 or 0, indicating normal movement in the positive direction, normal movement in the negative direction or backlash hysteresis, respectively. In Figure 12, the red dotted lines indicate the value of class label and the black solid lines indicate the value of the motor current. The value of class label is 15 or −15 when the end-effector stays in the stage of normal movement and 0 when the end-effector stays in the stage of backlash hysteresis. As shown in Figure 12, the designed classifier still performs well even though the movement patterns of the motor are different and the motor current fluctuates fiercely, which is the precondition of position compensation control.

### 3.3. Actual Angular Displacement Measurement and Position Compensation Control

**Figure 13:**
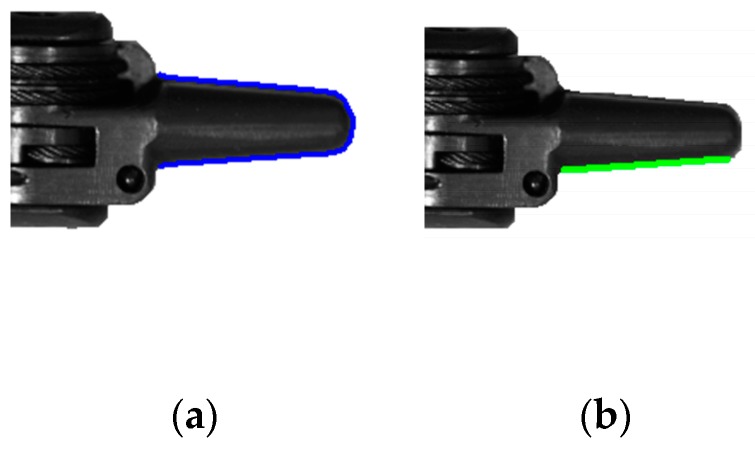
Edge extraction results of the images grabbed by the camera: (**a**) The sub-pixel precise edges of the end-effector’s tip; (**b**) The bottom edge of the end-effector’s tip.

**Figure 14:**
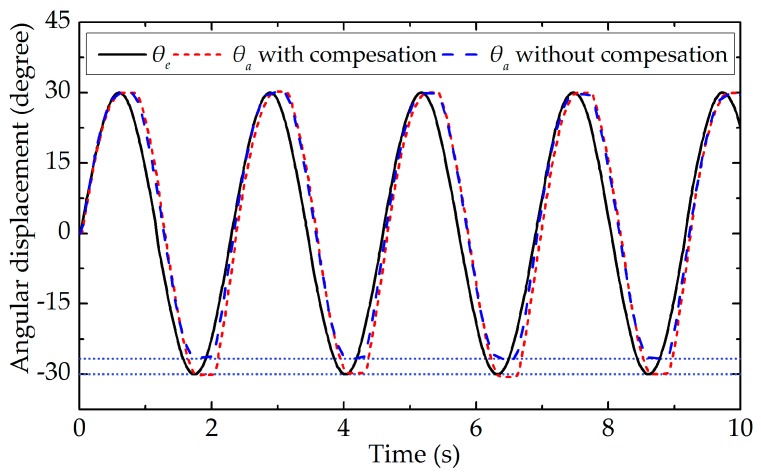
Comparison between the actual angular displacement of the end-effector with and without position compensation.

**Figure 15:**
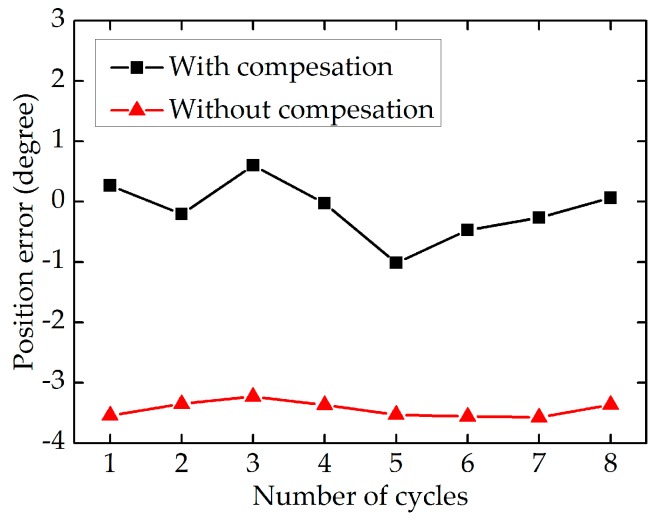
The position error of each cycle.

After the movement stage of the end-effector is classified accurately, the feedforward position compensation control scheme, which is illustrated in Figure 8, is used to reduce the tracking position errors. To obtain the actual angular displacement of the end-effector ($\theta_{a}$), the images of the end-effector grabbed by the camera are processed. As shown in Figure 13a, the sub-pixel precise edges of the end-effector’s tip are extracted, and as shown in Figure 13b, the bottom edge of the end-effector’s tip is used to calculate the actual angular displacement of the end-effector. To validate the effectiveness of the proposed position compensation scheme, the motor runs in the position control mode with a sinusoidal wave trajectory of $\theta_{c}=30\sin\left(2\pi ft\right){+\theta }_{f}$, where the frequency is equal to 0.47 Hz. Figure 14 shows the comparison of the actual angular displacement of the end-effector ($\theta_{a}$) with and without position compensation. We can see that $\theta_{a}$ with position compensation is closer to the expected angular displacement ($\theta_{e}$) from Figure 14. Figure 15 shows the position error of each cycle. It is obvious that tracking performance with compensation is improved significantly, with the mean absolute error decreasing from 3.441 degrees (without compensation) to 0.364 degrees (with compensation).

To compare the position compensation scheme presented in this paper with the methods of other studies, the position compensation effects of different schemes are listed in Table 3. The first row of Table 3 denotes some references cited in this paper, and Ref is the abbreviation of Reference. In the second row of Table 3, each data indicates the degree of positioning error reduction after adopting the position compensation scheme of the corresponding reference. For convenience, notation ERR denotes the error reduction rate.

It can be seen from Table 3 that the positioning error is reduced by 89.42% when the position compensation scheme presented in this paper is adopted, which verifies the effectiveness of the proposed scheme. Table 3 shows the proposed scheme has good performance when compared with the methods of other studies and can markedly improve the tracking performance of the system.

## 4. Discussion

In this paper, a novel feedforward position compensation control scheme is proposed to reduce the influence of backlash hysteresis during the surgical procedure. The theoretical basis of this article is that the kinetic characteristics of the driving motor are different when the end-effector is at different movement stages. General motion equations of the cable-pulley mechanism are derived under the assumption that the cable is equivalent to the spring damping model in which the bending and torsional moments of the cable are small enough to be neglected.

The premise of using the position compensation scheme is that the movement stages of the end-effector can be accurately classified by the classifier. As shown in Table 2 and Figure 12, the classification error rate of SVM is very small and the designed classifier has good classification performance under different motor motion patterns, which indicates the selection of features and the design of classifier are reasonable. As shown in Figure 14 and Figure 15, the actual and expected angular displacement of the end-effector are very close when the position compensation scheme is adopted, in contrast with the situation when it is not adopted. It is obvious that the tracking performance of the robotic end-effector can be improved significantly by using the proposed position compensation scheme, with the mean absolute error decreasing from 3.441 degrees (without compensation) to 0.364 degrees (with compensation). Table 3 shows the positioning error is reduced by 89.42% when the position compensation scheme presented in this paper is adopted. Compared with the methods of other studies, the proposed position compensation scheme has good performance.

Although many researchers have studied the nonlinear problems in cable-pulley mechanisms and provided appropriate solutions, lots of aspects of this field still need to be researched deeply and improved significantly. Existing studies mainly focus on designing transmission models under some assumptions to theoretically calculate the error between the actual and expected angular displacement. However, these approaches need a more complicated theoretical analysis and modeling process, and researchers should provide a good trade-off between the precision and complexity of the transmission model. In addition, the transmission model will change if the surgical instrument is replaced or the mechanical structure of the surgical instrument is changed, which frequently results in rebuilding the transmission model. In this paper, the proposed position compensation scheme resolves the problem of backlash hysteresis innovatively. This approach uses a classifier to recognize and classify different movement stages of the robotic end-effector and then compensates the position error based on feedforward control. The position compensation scheme proposed in this paper does not need to construct a complex transmission model and is easy to understand. If the surgical robot is reformed or replaced, we only need to re-collect examples and re-train the classifier. Another novelty of the manuscript is that the proposed strategy changed the traditional analysis pattern, which utilizes the machine learning algorithms to solve the backlash hysteresis problem caused by the mechanical structure. The experimental results show that the proposed position compensation scheme can efficiently improve the tracking performance and reduce the operation risk. The greater significance of this paper is that the proposed position compensation scheme provides a new method for improving the positioning accuracy of the robotic end-effector and is conducive to the motion control of the surgical robot.

In future work, we will improve the position compensation scheme for higher accuracy and add some parts that are missing in this research. In addition, we will research the coupling movement between different DOFs of the end-effector and try to find solutions.

## 5. Conclusions

This paper introduces a novel position compensation scheme to enhance the tracking performance of the robotic end-effector using cable-pulley mechanisms. Firstly, the kinetic model of a cable-pulley system used in the end-effector of a laparoscopic surgical robot was established to analyze the changes of motor driving torque. Then the movement stages of the end-effector were distinguished by the FFT result of the motor current. In order to improve the accuracy and robustness of the movement stage division, a classifier based on SVM was designed to classify the movement stage of the end-effector. Then, the feedforward position compensation control scheme was used to reduce the tracking position errors. Unlike the current compensation control approaches of the cable-driven system, our control scheme does not need to establish complex transmission models and is generally applicable to different cable-driven systems. Finally, an experimental setup with machine vision was designed to verify the effectiveness of the compensation control scheme. It has been demonstrated that the classifier based on SVM has better real-time performance and veracity under various motion patterns of the motor. The results of comparative experiments also prove that the proposed algorithm can improve the tracking performance of the cable-pulley system significantly. 

## Figures and Tables

**Table 1:** Mutual information of features.

Motor Direction	$F_{1}$	$F_{2}$	$F_{3}$	${\dot{\theta }}_{m}$	${\ddot{\theta }}_{m}$	$\theta_{er}$
Positive Movement	0.0290	0.0229	0.0173	0.0067	0.0166	0.0200
Negative Movement	0.0210	0.0162	0.0128	0.0055	0.0113	0.0125

**Table 2:** The prediction error rate of the classification.

Motor Direction	Train: B→N	Train: N→B	Test: B→N	Test: N→B
Positive Movement	0	0	0.069%	0.026%
Negative Movement	0.002%	0	0.039%	0.003%

**Table 3:** The position compensation effects of different schemes.

Reference	Ref [28]	Ref [23]	Ref [18]	This Paper	Ref [17]	Ref [25]
ERR	55.48%	67.14%	80.86%	89.42%	90.00%	93.94%
